# Valor Preditivo do Índice Aterogênico para Não-Refluxo em Pacientes Submetidos à ICP para Enxerto de Veia Safena na Síndrome Coronariana Aguda

**DOI:** 10.36660/abc.20250162

**Published:** 2025-12-18

**Authors:** Onur Erdoğan, Cafer Panç

**Affiliations:** 1 Department of Cardiology University of Health Sciences Istanbul Turquia Department of Cardiology, University of Health Sciences, Mehmet Akif Ersoy Thoracic and Cardiovascular Surgery Training and Research Hospital, Istanbul – Turquia

**Keywords:** Aterosclerose, Fenômeno de não Refluxo, Intervenção Coronária Percutânea, Síndrome Coronariana Aguda

## Abstract

**Fundamento:**

O fenômeno de não-refluxo é uma complicação crítica da intervenção coronária percutânea (ICP), especialmente em pacientes com doença do enxerto de veia safena (EVS). Identificar preditores de não-refluxo é crucial para melhorar a estratificação de risco. O índice aterogênico plasmático (IAP), calculado como log(triglicerídeos/HDL-C), é um marcador de aterosclerose e disfunção microvascular. No entanto, seu papel na predição de não-refluxo durante intervenções de EVS permanece incerto.

**Objetivo:**

Este estudo teve como objetivo avaliar a associação entre IAP e o fenômeno de não-refluxo em pacientes submetidos à ICP para lesões de EVS apresentando infarto do miocárdio sem supradesnivelamento do segmento ST (IAMSSST).

**Métodos:**

Este estudo retrospectivo de centro único incluiu 287 pacientes submetidos a ICP para estenose significativa do EVS. Os pacientes foram categorizados em grupos com refluxo normal e não-refluxo, com base nos graus de fluxo TIMI (trombólise no infarto do miocárdio) pós-ICP. Dados demográficos, clínicos, laboratoriais e angiográficos basais foram extraídos. O IAP foi calculado utilizando a fórmula logarítmica (TG/HDL-C). Análises de regressão logística foram realizadas para determinar os preditores independentes do não-refluxo. A significância estatística foi estabelecida em p < 0,05.

**Resultados:**

O grupo não-refluxo era mais velho e apresentava maior prevalência de comorbidades, incluindo diabetes, fibrilação atrial, insuficiência cardíaca crônica, doença renal crônica e histórico de acidente vascular cerebral. Eles também apresentaram níveis mais baixos de HDL-C, níveis mais altos de triglicerídeos e, consequentemente, valores mais altos de IAP em comparação ao grupo com refluxo normal. A análise multivariada identificou EVS degenerado (OR: 4,65, p < 0,001), presença de trombo (OR: 5,65, p < 0,001), fração de ejeção do ventrículo esquerdo reduzida (OR: 0,927, p < 0,001) e IAP elevado (OR: 3,141, p = 0,001) como preditores independentes de não-refluxo.

**Conclusão:**

O IAP elevado é um fator de risco independente para não-refluxo em pacientes com IAMSSST submetidos a ICP para EVS, destacando seu papel potencial na estratificação de risco precoce.

## Graphical abstract

**Figura Central:**
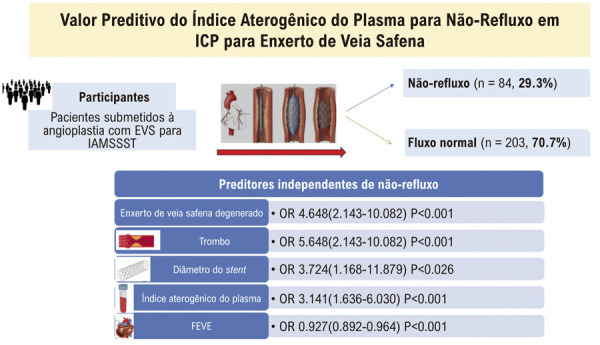
Valor Preditivo do Índice Aterogênico para Não-Refluxo em Pacientes Submetidos à ICP para Enxerto de Veia Safena na Síndrome Coronariana Aguda

## Introdução

As doenças cardiovasculares continuam sendo uma das principais causas de morbidade e mortalidade em todo o mundo, sendo a doença arterial coronariana (DAC) responsável por uma parcela substancial da carga global.^1^ A intervenção coronária percutânea (ICP) é um tratamento fundamental da DAC para alcançar a revascularização e melhorar a perfusão miocárdica.^2^ No entanto, o procedimento não é isento de complicações. Entre elas, o fenômeno de não-refluxo representa uma preocupação particularmente desafiadora e prognosticamente significativa tanto para médicos quanto para pacientes.

O não-refluxo é definido como a falha em atingir perfusão miocárdica adequada, apesar da revascularização bem-sucedida da artéria coronária,^3^ e está fortemente associado a desfechos hospitalares e de longo prazo desfavoráveis, incluindo infarto de maior tamanho, função ventricular esquerda reduzida e aumento da mortalidade. A fisiopatologia do não-refluxo é multifatorial, envolvendo disfunção microvascular, inflamação, disfunção endotelial, formação de trombos e vasoespasmo.^4^

Dessa forma, identificar marcadores pré-procedimentais que possam prever o não-refluxo é crucial para a estratificação de risco e a implementação de estratégias preventivas.

Nos últimos anos, tem crescido o interesse em preditores metabólicos e lipídicos de eventos cardiovasculares adversos. Entre eles, o Índice Aterogênico Plasmático (IAP) – definido como o logaritmo da razão entre triglicerídeos e HDL-colesterol – surgiu como um marcador substituto de dislipidemia aterogênica, resistência à insulina e disfunção endotelial. O IAP tem sido associado a risco cardiovascular aumentado, gravidade da DAC e complicações microvasculares.^5-7^ Refaat et al. demonstraram que níveis elevados de IAP estavam significativamente associados ao fenômeno de não-refluxo e a piores desfechos clínicos em pacientes com infarto do miocárdio com supradesnivelamento do segmento ST (IAMCSST) submetidos à ICP primária.^8^

Entretanto, apesar de sua relevância clínica, as evidências sobre o papel do IAP na previsão do não-refluxo ainda são limitadas, especialmente em subconjuntos de ICP de alto risco, como intervenções de enxerto de veia safena (EVS).

Os EVSs são comumente utilizados em procedimentos de revascularização do miocárdio (CRM). Com o tempo, os EVSs tendem a degenerar e apresentar predisposição à formação de trombos, o que aumenta o risco do fenômeno de não-refluxo em procedimentos de ICP direcionados a esses enxertos.

Embora se saiba que o não-refluxo ocorre com mais frequência em intervenções de EVS em comparação com artérias coronárias nativas,^9^ estudos explorando preditores confiáveis e acessíveis neste contexto específico estão ausentes.

Para abordar essa lacuna, o presente estudo tem como objetivo investigar a relação entre o IAP e o fenômeno de não-refluxo em pacientes com infarto do miocárdio sem supradesnivelamento do segmento ST (IAMSSST) submetidos à ICP para lesões de EVS. Nossa hipótese é que o IAP elevado, como marcador de estado metabólico pró-aterogênico e pró-inflamatório, pode servir como um preditor independente de não-refluxo nessa população de alto risco.

## Método

### Desenho do estudo e seleção de pacientes

Este estudo retrospectivo foi conduzido em um centro cardiovascular terciário de centro único. A população do estudo consistiu em 287 pacientes submetidos a ICP para doença do EVS com IAMSSST.

Foi utilizado um método de amostragem consecutiva, incluindo todos os pacientes elegíveis que atenderam aos critérios de inclusão entre fevereiro de 2018 e dezembro de 2022.

Os critérios de inclusão foram os seguintes: pacientes com histórico de CRM, pacientes com IAMSSST submetidos a ICP para estenose significativa de EVS e disponibilidade de dados de perfil lipídico pré-ICP, incluindo níveis de triglicerídeos e colesterol de lipoproteína de alta densidade (HDL).

Os critérios de exclusão incluíram pacientes com choque cardiogênico na apresentação, aqueles submetidos apenas à angioplastia percutânea com balão, implante prévio de *stent* no vaso índice, doença cardíaca valvar grave, reestenose ou trombose de *stent*, ICP para IAMCSST ou ICP para lesões de artéria coronária nativa em vez de EVS.

O estudo foi conduzido de acordo com os princípios da Declaração de Helsinque e foi aprovado pelo Comitê de Ética da Universidade de Istambul, Faculdade de Medicina de Istambul (2025.01-06). O consentimento informado por escrito foi obtido de todos os participantes incluídos no estudo.

***Determinação do tamanho da amostra***: Em um estudo conduzido por Wu et al., que teve como objetivo investigar a relação entre o IAP e o risco de DAC, bem como determinar se o IAP é um melhor preditor de risco de DAC em mulheres na pós-menopausa, o IAP foi relatado como 0,20 ± 0,27 no grupo DAC e 0,10 ± 0,27 no grupo sem DAC.^10^ Com base neste estudo, foi assumido que uma diferença semelhante (d de Cohen = ~0,3, representando um tamanho de efeito pequeno a médio) também poderia ser observada entre os grupos não-refluxo e não-não-refluxo em nosso estudo. Sob essas suposições, uma análise de poder determinou que um mínimo de 114 pacientes por grupo, para um total de 228 pacientes, seria necessário para atingir 80% de poder com uma margem de erro de 5%.

Todos os dados demográficos, clínicos e laboratoriais iniciais foram obtidos de registros médicos eletrônicos.

### Angiografia coronária e procedimento de ICP

A angiografia coronária e os procedimentos de ICP foram realizados de acordo com os protocolos institucionais padrão. Todos os pacientes receberam terapia antiplaquetária dupla, incluindo 300 mg de aspirina e um inibidor P2Y12 (clopidogrel 600 mg, ticagrelor 180 mg ou prasugrel 60 mg) e 80 mg de atorvastatina antes da intervenção. Uma dose em bolus de heparina de 70–100 UI/kg foi administrada a cada paciente agendado para angioplastia.

Estratégias de ICP, incluindo seleção de *stent*, uso de pré-dilatação ou pós-dilatação, dispositivos de proteção distal ou trombectomia, ficaram a critério do operador. Os graus de fluxo TIMI (trombólise no infarto do miocárdio) foram avaliados antes e depois do procedimento para classificar os pacientes em grupos não-refluxo ou refluxo normal. O sistema de classificação TIMI define grau 0 como a ausência de fluxo anterógrado além da oclusão, enquanto o grau 1 indica fluxo distal mínimo que não consegue opacificar completamente a artéria. O grau 2 representa opacificação tardia, mas completa, do vaso distal, enquanto o grau 3 reflete perfusão normal com fluxo irrestrito. O não-refluxo foi diagnosticado quando o fluxo TIMI pós-ICP permaneceu abaixo do grau 3 na ausência de obstrução mecânica, como dissecção, estenose residual ou vasoespasmo.

Após o implante do *stent*, os pacientes foram inicialmente classificados de acordo com seus graus de fluxo TIMI imediatamente após o implante do *stent*. Em casos de não-refluxo, terapias farmacológicas intracoronárias, incluindo inibidores de adenosina e glicoproteína IIb/IIIa, epinefrina ou vasodilatadores, foram aplicadas a critério do operador. Os graus de fluxo TIMI final foram reavaliados após essas intervenções, e os pacientes que atingiram fluxo TIMI 3 foram registrados adequadamente. Em oito pacientes, essas intervenções restauraram com sucesso o fluxo TIMI 3 final. No entanto, para análise estatística e classificação do grupo, o grau de fluxo TIMI avaliado imediatamente após o implante do *stent* e antes de intervenções farmacológicas adicionais foi usado. Portanto, o grupo não-refluxo inclui pacientes independentemente de qualquer melhora posterior no fluxo TIMI após o tratamento.

### Medição de parâmetros lipídicos

Amostras de sangue foram coletadas após jejum noturno antes da intervenção e analisadas quanto aos parâmetros lipídicos por meio de ensaios enzimáticos automatizados. O colesterol total, o colesterol HDL, o colesterol de lipoproteína de baixa densidade (LDL-C) e os triglicerídeos (TG) foram dosados em um autoanalisador Abbott Diagnostics C8000i (Abbott, Alemanha) com kits comerciais. O IAP foi calculado pela fórmula: log (TG/HDL-C).

### Análise estatística

Todas as análises estatísticas foram realizadas usando SPSS software Versão 21.0 para Windows (SPSS Inc., Chicago, IL, EUA). A normalidade da distribuição dos dados foi avaliada pelo teste de Shapiro-Wilk. As variáveis contínuas foram expressas como média ± desvio-padrão (DP) para dados com distribuição normal e como mediana e intervalo interquartil (IIQ) para dados com distribuição anormal. As variáveis categóricas foram apresentadas como frequências e porcentagens.

Comparações entre os grupos não-refluxo e com refluxo normal foram conduzidas usando os seguintes testes: teste-t para amostras independentes para variáveis contínuas com distribuição normal, teste U de Mann-Whitney para variáveis contínuas com distribuição não normal, teste chi-quadrado ou teste exato de Fisher para variáveis categóricas. Uma análise de regressão logística univariada foi conduzida para identificar potenciais preditores do fenômeno não-refluxo. Modelos de regressão logística multivariável foram inicialmente construídos usando variáveis estatisticamente significativas (p < 0,05) em análises univariadas. A multicolinearidade entre os preditores foi avaliada antes da inclusão do modelo, e variáveis com colinearidade significativa foram excluídas. Para reduzir o risco de sobreajuste, uma análise de sensibilidade foi realizada usando um modelo parcimonioso compreendendo os principais preditores selecionados com base na relevância clínica e na força univariada. Os resultados foram relatados como razões de chances (ORs) com intervalos de confiança (ICs) de 95%. Um valor de p < 0,05 foi considerado estatisticamente significativo para todas as análises.

## Resultados

Um total de 287 pacientes submetidos a ICP para doença do EVS foram incluídos no estudo. Esses pacientes foram categorizados em dois grupos: refluxo normal (70,7%) e não-refluxo (29,3%). As características demográficas, clínicas e laboratoriais basais da população do estudo estão resumidas na Tabela 1.

**Tabela 1:** – As características demográficas, clínicas e laboratoriais basais da população do estudo

Variáveis	Não-refluxo (-) (n: 203)	Não-refluxo (+) (n: 84)	Valor de p	% Diferença (Não-refluxo - Refluxo)
Idade (anos)	62,6±8,6	66,1±10	0,003	
Sexo masculino, n (%)	173 (85,2%)	67 (79,8%)	0,255	
Fumante atual, n (%)	44 (21,7%)	48 (57,1%)	0,061	
Hipertensão, n (%)	155 (76,4%)	71 (84,5%)	0,124	
Diabetes mellitus, n (%)	80 (39,4%)	48 (57,1%)	0,006	17,17%
Dislipidemia, n (%)	117 (57,6%)	48 (57,1%)	0,939	
Fibrilação atrial, n (%)	19 (9,4%)	15 (17,9%)	0,043	8,5%
Insuficiência cardíaca crônica, n (%)	39 (19,2%)	52 (61,9%)	<0,001	42,7%
Doença renal crônica, n (%)	47 (23,2%)	31 (36,9%)	0,017	13,7%
Doença arterial periférica, n (%)	41 (20,2%)	25 (29,8%)	0,080	
DPOC, n (%)	28 (13,8%)	9 (10,7%)	0,479	
Histórico de acidente vascular cerebral/AIT, n (%)	10 (4,9%)	19 (22,6%)	<0,001	17,7%
IM anterior, n (%)	97 (47,8%)	52 (61,9%)	0,029	14,1%
ICP anterior, n (%)	74 (36,5%)	39 (46,4%)	0,116	
FEVE, (%)	55 (45-60)	45 (35-50)	<0,001	
Hemoglobina (g/dl)	13,5±1,8	12,5±1,9	<0,001	
Colesterol total (mg/dl)	192±54,5	202±57,3	0,075	
HDL-c (mg/dl)	39,7±9,2	35,3±9,9	0,001	
LDL-c, (mg/dl)	115,2±45,7	116±53,2	0,961	
Triglicerídeos (mg/dl)	163 (114-241)	205 (124-408)	0,002	
IAP	1,4 (1,04-2,00)	1,9 (1,23-2,50)	<0,001	

As variáveis com distribuição normal foram expressas como média±DP e comparadas com o teste-t de Student, e variáveis contínuas sem distribuição normal são apresentadas como mediana (intervalo interquartil, IIQ) e comparadas com o teste U de Mann–Whitney. IAP: índice aterogênico do plasma; DPOC: doença pulmonar obstrutiva crônica; HDL-c: colesterol de lipoproteína de alta densidade; LDL-c: colesterol de lipoproteína de baixa densidade; FEVE: fração de ejeção do ventrículo esquerdo; IM: infarto do miocárdio; ICP: intervenção coronária percutânea; AIT: ataque isquêmico transitório.

### Características de base

Em comparação com o grupo com refluxo normal, os pacientes do grupo não-refluxo eram mais velhos e apresentavam maior carga de comorbidades, incluindo diabetes, fibrilação atrial, doença renal crônica, insuficiência cardíaca crônica e AVC ou ataque isquêmico transitório (AIT) prévios. Análises laboratoriais revelaram níveis mais baixos de HDL-C e níveis mais altos de triglicerídeos no grupo não-refluxo, resultando em um IAP significativamente elevado.

### Características angiográficas e processuais

As características angiográficas e do procedimento estão detalhadas na Tabela 2. A incidência de EVS degenerado foi significativamente maior no grupo não-refluxo. Além disso, o uso de *stent*s com diâmetros maiores foi mais comum no grupo não-refluxo, enquanto o comprimento do *stent* não diferiu significativamente (p = 0,559).

**Tabela 2:** – As características angiográficas e processuais coronárias dos pacientes

Variáveis	Refluxo normal	Não-refluxo	Valor de p	% Diferença
Intervalo entre a operação e a angiografia (anos)	10.11 (5.06)	11,12 (4,98)	0,120	
Enxerto de veia safena estreitada para			0,910	
ADAE, n (%)	13 (6,4%)	7 (8,3%)		
Diagonal, n (%)	22 (10,8%)	9 (10,7%)		
CX, n (%)	86 (42,24%)	37 (44%)		
ACD, n (%)	82 (40,4%)	31 (36,9%)		
Local da lesão			0,074	
Óstio, n (%)	22 (10,8%)	15 (17,9%)		
Proximal, n (%)	55 (27,1%)	27 (32,1%)		
Médio, n (%)	63 (31%)	22 (26,2%)		
Distal, n (%)	53 (26,1%)	12 (14,3%)		
Grau de fluxo TIMI pré-intervenção			<0,001	
TIMI 0, n (%)	19 (9,4%)	22 (26,2%)		
TIMI 1, n (%)	14 (6,9%)	8 (9,5%)		
TIMI 2, n (%)	53 (26,1%)	30 (35,7%)		
TIMI 3, n (%)	117 (57,6%)	24 (28,6%)		
Grau de fluxo TIMI pós-intervenção				
TIMI 0, n (%)	0 (0%)	7 (8,3%)	<0,001	
TIMI 1, n (%)	0 (0%)	20 (23,8%)	<0,001	
TIMI 2, n (%)	0 (0%)	49 (58,3%)	<0,001	
TIMI 3, n (%)	203 (100%)	8 (9,5%)	<0,001	
Enxerto de veia safena degenerada, n (%)	45 (22,2%)	53 (63,1%)	<0,001	40,9%
Trombo, n (%)	40 (19,7%)	55 (65,5%)	<0,001	45,8%
Diâmetro do *stent* (mm)	3,19 (0,51)	3,46 (0,59)	0,001	
Comprimento do *stent* (mm)	24,1 (11,81)	25,54 (13,59)	0,559	
Pré-dilatação, n (%)	71 (35%)	38 (45,2%)	0,083	
Pós-dilatação, n (%)	48 (23,6%)	14 (16,7%)	0,191	
Uso de ACO, n (%)	15 (7,4%)	8 (9,5%)	0,544	
Uso de inibidor da glicoproteína IIb/IIIa, n (%)	35 (17,2%)	41 (48,8%)	<0,001	31,6%
Uso de dispositivo de proteção distal, n (%)	8 (3,9%)	4 (4,8%)	0,751	
Trombectomia, n (%)	2 (1%)	4 (4,8%)	0,063	

CX: artéria circunflexa; ADAE: artéria descendente anterior esquerda; ACD: artéria coronária direita; ACO: anticoagulante oral; TIMI: trombólise no infarto do miocárdio.

### Preditores de não-refluxo

Análises de regressão logística univariada e multivariada foram realizadas para identificar preditores de não-refluxo (Tabela 3). Na análise univariada, idade, diabetes mellitus, doença renal crônica, insuficiência cardíaca crônica, histórico de acidente vascular cerebral/AIT, fibrilação atrial, baixa fração de ejeção do ventrículo esquerdo (FEVE), presença de EVS degenerado, carga trombótica, diâmetro do *stent* e relação triglicerídeos/HDL foram significativamente associados ao não-refluxo.

**Tabela 3:** – Análise de regressão logística univariada e multivariada dos preditores do fenômeno de não-refluxo

	Análises univariadas		Análises multivariadas	
Variáveis	OR (IC 95%)	Valor-p	OR (IC 95%)	Valor-p
Idade	1,044 (1,014-1,075)	0,004	1,032 (0,986-1,080)	0,176
Sexo masculino	0,683 (0,354-1,320)	0,257		
Hipertensão	1,691 (0,862-3,319)	0,127		
Diabetes Mellitus	2.050 (1.224-3.433)	0,006	2,127 (0,990-4,568)	0,053
Dislipidemia	0,980 (0,586-1,639)	0,939		
Fumante atual	1,712 (0,971-3,017)	0,063		
Doença renal crônica	1,941 (1,120-3,366)	0,018	0,692 (0,297-1,613)	0,393
Insuficiência cardíaca crônica	6.883 (3.895-11.987)	<0,001		
Doença arterial periférica	1,674 (0,938-2,990)	1.674		
DPOC	0,750 (0,338-1,666)	0,480		
Histórico de acidente vascular cerebral/AIT	5.642 (2.495-12.754)	<0,001	2,986 (0,999-8,925)	0,050
Fibrilação atrial	2,105 (1,013-4,374)	0,046	2,404 (0,743-7,775)	0,143
IM anterior	1,776 (1,056-2,985)	0,030	1,114 (0,500-2,485)	0,792
ICP anterior	1,511 (0,902-2,529)	0,110		
FEVE	0,916 (0,891-0,942)	<0,001	0,927 (0,892-0,964)	<0,001
Intervalo entre a operação e a angiografia	1,040 (0,987-1,096)	0,140		
EVS degenerado	6.320 (3.625-11.017)	<0,001	4.648 (2.143-10.082)	<0,001
Trombo	8.150 (4.607-14.418)	<0,001	5.648 (2.143-10.082)	<0,001
Diâmetro do *stent*	2.387 (1.467-3.885)	<0,001	3.724 (1.168-11.879)	0,026
Comprimento do *stent*	1,009 (0,998-1,031)	0,393		
Pré-dilatação	1,577 (0,941-2,642)	0,084		
Pós-dilatação	0,646 (0,334-1,248)	0,193		
Tipo de *stent*	0,526 (0,307-0,901)	0,019	1,520 (0,424-5,450)	0,521
IAP	2.332 (1.608-3.382)	<0,001	3.141 (1.636-6.030)	0,001

OR Odds ratios; IC: intervalos de confiança; IAP: índice aterogênico do plasma; DPOC: doença pulmonar obstrutiva crônica; FEVE: fração de ejeção do ventrículo esquerdo; IM: infarto do miocárdio; ICP: intervenção coronária percutânea; EVS: enxerto de veia safena; AIT: ataque isquêmico transitório.

Na análise de regressão logística multivariada, EVS degenerado (OR: 4,648, IC 95%: 2,143–10,082, p < 0,001), presença de trombo (OR: 5,648, IC 95%: 2,143–11,879, p < 0,001), FEVE reduzida (OR: 0,927, IC 95%: 0,892–0,964, p < 0,001), diâmetro de *stent* maior (OR: 3,724, IC 95%: 1,168-11,879, p: 0,026) e IAP elevado (OR: 3,141, IC 95%: 1,636–6,030, p = 0,001), permaneceram preditores independentes do fenômeno de não-refluxo. A figura central resume os principais achados descritos acima. Para abordar ainda mais o risco de sobreajuste, uma análise de sensibilidade foi conduzida repetindo a regressão logística multivariável com as seis principais variáveis com base em sua força univariada e relevância clínica (EVS degenerado, trombo, FEVE reduzida, IAP e histórico de AVC/AIT). Este modelo parcimonioso produziu resultados consi*stent*es, reafirmando a robustez de nossos achados (Tabela 4).

**Tabela 4:** – Análise de Sensibilidade dos Preditores Independentes do Fenômeno de Não-Refluxo

Análises multivariadas		
Variáveis	OR (IC 95%)	Valor-p
Histórico de acidente vascular cerebral/AIT	3.130 (1.102-8.889)	0,032
FEVE	0,921 (0,888-0,954)	<0,001
EVS degenerado	4.814 (2.325-9.970)	<0,001
Trombo	4.978 (2.369-10.460)	<0,001
Diâmetro do stent	2.460 (1.291-4.686)	0,006
IAP	2.531 (1.451-4.414)	0,001

OR: odds ratio; IC: intervalo de confiança; IAP: índice aterogênico do plasma; FEVE: fração de ejeção do ventrículo esquerdo; EVS: enxerto de veia safena; AIT: ataque isquêmico transitório.

## Discussão

Neste estudo, investigamos a relação entre o fenômeno de não-refluxo que ocorre durante a intervenção com EVS em pacientes com IAMSSST com histórico de CRM e o IAP, calculado como log (razão triglicerídeos/HDL). Nossa análise multivariada demonstrou que o EVS degenerado, a presença de trombo, a FEVE reduzida e o IAP elevado foram preditores independentes de NR. A facilidade de obtenção da razão triglicerídeos/HDL na prática clínica de rotina sugere que este parâmetro pode servir como um marcador útil para prever a disfunção microvascular e o risco de não-refluxo.

Nossos achados são consi*stent*es com outros estudos na literatura que destacam a associação do IAP com o risco de DAC, progressão da placa e o mecanismo de não-refluxo impulsionado por lesão microvascular. Estudos conduzidos em pacientes com IAMCSST demonstraram que o IAP prediz o não-refluxo de forma independente e oferece poder preditivo superior em comparação com outros parâmetros lipídicos.^11-13^ O cálculo relativamente simples do IAP e de parâmetros lipídicos semelhantes em ambientes clínicos reforça ainda mais a importância deste indicador na estimativa do risco de não-refluxo.

O IAP é calculado como o logaritmo da razão entre triglicerídeos (TG) e HDL-C, servindo como um importante biomarcador de processos ateroscleróticos na parede vascular. Surgiu como uma ferramenta promissora para avaliar o risco de DAC, a gravidade da doença e o prognóstico.^11^ Estudos indicam que o IAP está fortemente associado a múltiplos mecanismos pró-aterogênicos, incluindo lipoproteínas remanescentes elevadas, aumento da esterificação do colesterol e resistência à insulina, que juntos promovem disfunção endotelial, vulnerabilidade da placa e um estado pró-inflamatório — principais contribuintes para o fenômeno de não-refluxo.^14-16^ O IAP elevado reflete um desequilíbrio entre lipoproteínas ricas em triglicerídeos aterogênicas e partículas protetoras de HDL. Esse desequilíbrio promove a formação de partículas de LDL pequenas e densas, que são mais propensas à oxidação, levando à lesão endotelial e à inflamação. Além disso, níveis elevados de triglicerídeos contribuem para uma maior carga de lipoproteínas remanescentes, que se infiltram na íntima vascular e estimulam respostas inflamatórias locais. Esses processos não apenas aumentam a progressão da aterosclerose, mas também aumentam o risco de embolização distal e dano microvascular. Além disso, baixos níveis de HDL reduzem o transporte reverso de colesterol e o mecanismo de reparo endotelial, comprometendo ainda mais a microcirculação coronária. Coletivamente, esses mecanismos interrelacionados oferecem uma explicação fisiopatológica clara de como o IAP elevado pode predispor os pacientes ao não-refluxo durante a ICP.

Pacientes com histórico de CRM são explicitamente classificados como de risco cardiovascular muito alto, o que recomenda uma meta de LDL-C < 55 mg/dL (1,4 mmol/L) e uma redução ≥ 50% em relação ao valor basal. Em casos de um segundo evento vascular em até dois anos, uma meta mais rigorosa de < 40 mg/dL (1,0 mmol/L) pode ser considerada.^17^ Evidências genéticas, epidemiológicas e de ensaios clínicos randomizados robustos estabeleceram uma relação causal direta entre LDL-C elevado e doença cardiovascular aterosclerótica, incluindo falência do enxerto.^18^ Consequentemente, a terapia hipolipemiante intensiva, principalmente com altas doses de estatinas e agentes adjuvantes, como ezetimiba ou inibidores de PCSK9, é essencial para a prevenção secundária.^17,19,20^ Em nossa coorte de estudo, os níveis médios de LDL-C permaneceram acima da meta, apesar da CRM prévia, sugerindo intensidade de tratamento abaixo do ideal ou baixa adesão. Essa carga aterogênica persi*stent*e pode ter contribuído para a perfusão microvascular prejudicada e o aumento do risco de desfechos adversos. Esses achados destacam a importância crucial de atingir as metas lipídicas recomendadas pelas diretrizes no contexto da prevenção secundária, particularmente entre pacientes submetidos à revascularização cirúrgica.

No contexto de doenças crônicas cardiometabólicas, o metabolismo lipídico prejudicado e a resistência à insulina desempenham papéis importantes na aterogênese, enfatizando o valor de indicadores baseados em lipoproteínas, como o IAP, na avaliação de risco cardiometabólico.^14^ Outra investigação que examinou a relação entre IAP, resistência à insulina e diabetes tipo 2 revelou uma associação não linear, sugerindo que o IAP elevado pode ser um marcador de risco importante, especialmente para diabetes e resistência à insulina.^15^ Além disso, altos níveis de IAP foram relatados como aumentando as lipoproteínas remanescentes e a esterificação do colesterol, acelerando assim as alterações aterogênicas na parede vascular.^21^ Coletivamente, essas descobertas demonstram que o IAP está fortemente associado à resistência à insulina, à lipoproteinemia remanescente e ao aumento da esterificação do colesterol — fatores que aceleram a aterosclerose — e dão suporte ao seu uso eficaz na prática clínica para avaliação de risco cardiometabólico.

O não-refluxo surge de alterações funcionais e estruturais na microcirculação coronária, explicadas por quatro mecanismos principais: embolização distal, lesão isquêmica, lesão de reperfusão e suscetibilidade individual.^22^ Especificamente, a embolização de material de placas ateroscleróticas no leito vascular distal promove microinfartos e inflamação, facilitando o não-refluxo.^23^

A isquemia prolongada aumenta a morte celular e o edema, ao mesmo tempo que reduz a produção de óxido nítrico, o que aumenta ainda mais a permeabilidade vascular através do fator de crescimento endotelial vascular (VEGF).^24^

A lesão por reperfusão agrava o dano microvascular por meio da infiltração de neutrófilos, liberação de citocinas e estresse oxidativo. Disfunção endotelial preexi*stent*e ou predisposições genéticas também podem aumentar a suscetibilidade ao não-refluxo.

A inflamação desempenha um papel particularmente crítico nesse processo, pois é amplamente reconhecida como um fator-chave tanto para a fisiopatologia da DAC quanto para o desenvolvimento de não-refluxo. Vários estudos demonstraram que níveis mais elevados de proteína C-reativa (PCR) e concentrações mais baixas de HDL-C estão associados ao não-refluxo, destacando o impacto proeminente de um estado inflamatório nesse fenômeno.^25^ Revisões sistemáticas também indicam que as partículas remanescentes produzidas durante a lipólise de lipoproteínas ricas em triglicerídeos podem provocar inflamação vascular, avançando assim a aterosclerose.^26^ Portanto, a maior incidência de não-refluxo observada em pacientes com IAP elevado pode ser amplamente atribuída a processos inflamatórios subjacentes. Como o IAP pode ser facilmente medido na prática de rotina, ele se mostra promissor como um indicador prático para prever o risco de não-refluxo associado à disfunção microvascular e à inflamação.

Em nosso estudo, além do IAP elevado, constatamos que a degeneração do EVS, a presença de trombo, o diâmetro do *stent* e a redução da FEVE surgiram como preditores independentes do fenômeno de não-refluxo. Enxertos degenerados provavelmente refletem alterações ateroscleróticas avançadas e maior vulnerabilidade da placa, predispondo à embolização distal e obstrução microvascular — um achado que se alinha com relatos anteriores que demonstram o impacto adverso da degeneração do enxerto nos desfechos de reperfusão. Da mesma forma, a formação de trombo dentro do enxerto pode levar à embolização distal, microinfartos e subsequente comprometimento da perfusão microvascular, conforme também destacado por outros estudos em cenários clínicos semelhantes. A redução da FEVE, por outro lado, não apenas indica a gravidade do dano miocárdico, mas também sugere uma reserva microcirculatória prejudicada, aumentando, assim, o risco de não-refluxo. Essas associações reforçam a natureza multifatorial do fenômeno de não-refluxo e a importância de integrar parâmetros lipídicos e hemodinâmicos na estratificação de risco, conforme corroborado pela literatura.^8^

Além disso, nossos achados revelaram que um diâmetro maior do *stent* foi independentemente associado ao não-refluxo. Uma possível explicação é que *stent*s maiores são frequentemente necessários para enxertos de veia safena gravemente degenerados ou ectásicos, que tendem a apresentar maior carga trombótica e placas ateroscleróticas friáveis. Essas características anatômicas aumentam o risco de embolização distal e obstrução microvascular, mecanismos-chave que contribuem para o desenvolvimento do não-refluxo. Essa observação é consi*stent*e com relatos anteriores que indicam que a complexidade da lesão e a carga de placa são fatores críticos para o comprometimento da perfusão microvascular durante ICP em lesões de EVS.^22,24^

Limitações e Pontos Fortes: Apesar desses insights promissores, nosso estudo apresenta certas limitações. O desenho retrospectivo e a natureza unicêntrica podem restringir a generalização dos achados, e potenciais fatores de confusão inerentes à pesquisa observacional não puderam ser totalmente descartados. No entanto, os pontos fortes do estudo incluem a avaliação abrangente de parâmetros clínicos e angiográficos rotineiramente disponíveis, o que aumenta sua aplicabilidade na prática diária. Outra limitação do nosso estudo é o uso relativamente baixo de dispositivos de proteção distal durante intervenções de EVS, apesar das recomendações das diretrizes que su*stent*am seu benefício em casos selecionados. Isso pode ter contribuído para a alta incidência de não-refluxo, permitindo embolização distal mais frequente. O uso limitado desses dispositivos deveu-se, em grande parte, a restrições locais de saúde e barreiras relacionadas a custos em nosso país. Embora as diretrizes da ACC/AHA recomendem dispositivos de proteção embólica (DPEs) para intervenções de EVS (indicação de Classe I), o uso no mundo real permanece baixo. Dados do NCDR Cath Registry (~21% de uso) não mostraram redução significativa nos desfechos adversos e até mesmo complicações processuais mais elevadas, como não-refluxo e perfuração, no grupo DPE. Esses achados destacam que o uso de DPE deve ser individualizado com base na carga trombótica, complexidade da lesão e experiência do operador.^27^ Em nosso centro, restrições de custo e disponibilidade limitada restringiram ainda mais o uso de DPE a casos selecionados de alto risco. Além disso, embora todos os pacientes tivessem histórico de revascularização coronária prévia, os níveis médios de LDL-C em nossa coorte permaneceram acima da meta recomendada de 50 mg/dL, particularmente para prevenção secundária. Esse controle lipídico subótimo pode refletir subutilização ou não adesão à terapia intensiva com estatinas e pode ter influenciado a disfunção microvascular e o risco de não-refluxo. Outra limitação é a ausência de correspondência por escore de propensão (PSM) para ajustar as diferenças basais. Devido ao número limitado de casos de não-refluxo, a aplicação do PSM poderia ter reduzido significativamente o tamanho da amostra e o poder estatístico. Estudos futuros com populações maiores devem considerar o uso de PSM ou outras técnicas avançadas de correspondência para validar essas descobertas.

Além disso, ao confirmar o valor preditivo independente de marcadores facilmente obtidos, como IAP, enxertos degenerados, presença de trombo e FEVE, nosso trabalho contribui com evidências valiosas para melhorar a avaliação de risco e orientar estratégias preventivas para o fenômeno de não-refluxo em populações de pacientes de alto risco.

Estudos futuros devem, idealmente, adotar delineamentos prospectivos e multicêntricos para validar nossos achados e explorar potenciais intervenções que possam mitigar esses fatores de risco. Investigar estratégias para melhorar a integridade do enxerto e reduzir a carga trombótica pode ser particularmente valioso. Além disso, avaliar o valor prognóstico da IAP em populações mais amplas de pacientes e determinar valores de corte ideais para uso clínico seria benéfico para refinar a estratificação de risco e adaptar as abordagens de tratamento.

Dada a sua simplicidade e acessibilidade, o IAP pode servir como um marcador prognóstico útil não apenas em intervenções de EVS, mas também em populações mais amplas, como aquelas com doença arterial coronária nativa ou síndromes coronárias crônicas. Mais estudos prospectivos são necessários para validar seu papel e estabelecer valores de corte clinicamente significativos nesses cenários.

## Conclusão

Em resumo, nosso estudo demonstra que IAP elevado, EVSs degenerados, presença de trombo, diâmetro do *stent* e FEVE reduzida estão independentemente associados ao fenômeno de não-refluxo em uma população de pacientes cuidadosamente selecionada. Esses achados fornecem insights adicionais sobre a natureza multifatorial do não-refluxo e sugerem que parâmetros clínicos e angiográficos rotineiramente disponíveis podem auxiliar em sua identificação precoce. No entanto, dado o desenho retrospectivo e unicêntrico do nosso estudo, pesquisas adicionais são necessárias para confirmar essas associações e explorar o impacto de intervenções direcionadas na melhoria dos desfechos clínicos em pacientes de alto risco.
