# Supplementary material for: Valor Preditivo do Índice Aterogênico para Não-Refluxo em Pacientes Submetidos à ICP para Enxerto de Veia Safena na Síndrome Coronariana Aguda
Source: Arq Bras Cardiol. 2025 Dec 18;122(11):e20250162. [Article in Portuguese] doi: 10.36660/abc.20250162 (PMC12978274; doi:10.36660/abc.20250162)
Supplement: Translation

# Translation: Predictive Value of Atherogenic Index for No-Reflow in Patients Undergoing Coronary Intervention for Saphenous Vein Graft with Acute Coronary Syndrome

**DOI:** 10.36660/abc.20250162i

**Authors:** Onur Erdoğan, Cafer Panç

**Affiliations:** 1 Turkiye Department of Cardiology, University of Health Sciences, Mehmet Akif Ersoy Thoracic and Cardiovascular Surgery Training and Research Hospital, Istanbul - Turkiye

**Keywords:** Atherosclerosis, No-Reflow Phenomenon, Percutaneous Coronary Intervention, Acute Coronary Syndrome

## Abstract

**Background:**

The no-reflow phenomenon is a critical complication of percutaneous coronary intervention (PCI), especially in patients with saphenous vein graft (SVG) disease. Identifying predictors of no-reflow is crucial to improving risk stratification. The atherogenic index of plasma (AIP), calculated as log(triglyceride/HDL-C), is a marker of atherosclerosis and microvascular dysfunction. However, its role in predicting no-reflow during SVG interventions remains unclear.

**Objective:**

This study aimed to evaluate the association between AIP and the no-reflow phenomenon in patients undergoing PCI for SVG lesions presenting with non-ST elevation myocardial infarction (NSTEMI).

**Methods:**

This single-center retrospective study included 287 patients who underwent PCI for significant SVG stenosis. Patients were categorized into normal reflow and no-reflow groups based on post-PCI TIMI (Thrombolysis in Myocardial Infarction) flow grades. Baseline demographic, clinical, laboratory, and angiographic data were extracted. AIP was calculated using formula log (TG/HDL-C). Logistic regression analyses were performed to determine independent predictors of the no-reflow. Statistical significance was set at p<0.05.

**Results:**

The no-reflow group was older and had a higher prevalence of comorbidities, including diabetes, atrial fibrillation, chronic heart failure, chronic kidney disease, and stroke history. They also exhibited lower HDL-C levels, higher triglyceride levels, and consequently higher AIP values compared to the normal reflow group. Multivariate analysis identified degenerated SVG (OR: 4.65, p<0.001), thrombus presence (OR: 5.65, p<0.001), reduced left ventricular ejection fraction (OR: 0.927, p<0.001), and elevated AIP (OR: 3.141, p=0.001) as independent predictors of the no-reflow.

**Conclusion:**

Elevated AIP is an independent risk factor for no-reflow in NSTEMI patients undergoing PCI for Saphenous Vein Graft, highlighting its potential role in early risk stratification.

## Introduction

Cardiovascular diseases remain a leading cause of morbidity and mortality worldwide, with coronary artery disease (CAD) accounting for a substantial portion of the global burden.^1^ Percutaneous coronary intervention (PCI) is a cornerstone treatment of CAD to achieve revascularization and enhance myocardial perfusion.^2^ However, the procedure is not without complications. Among them, the no-reflow phenomenon represents a particularly challenging and prognostically significant concern for both clinicians and patients.

No-reflow is defined as the failure to achieve adequate myocardial perfusion despite successful coronary artery revascularization,^3^ and is strongly associated with poor in-hospital and long-term outcomes, including larger infarct size, reduced left ventricular function, and increased mortality. The pathophysiology of no-reflow is multifactorial, involving microvascular dysfunction, inflammation, endothelial dysfunction, thrombus formation, and vasospasm.^4^

As such, identifying preprocedural markers that could predict no-reflow is crucial for risk stratification and the implementation of preventive strategies.

In recent years, interest has grown in metabolic and lipid-related predictors of adverse cardiovascular events. Among them, the Atherogenic Index of Plasma (AIP) -defined as the logarithm of the triglyceride to HDL-cholesterol ratio- has emerged as a surrogate marker of atherogenic dyslipidemia, insulin resistance, and endothelial dysfunction. AIP has been linked with increased cardiovascular risk, coronary artery disease severity, and microvascular complications.^5-7^ Refaat et al. demonstrated that elevated AIP levels were significantly associated with the no-reflow phenomenon and worse clinical outcomes in patients with ST-elevation myocardial infarction (STEMI) undergoing primary PCI. ^8^

However, despite its clinical relevance, evidence regarding the role of AIP in predicting no-reflow is still limited, especially in high-risk PCI subsets such as saphenous vein graft (SVG) interventions.

SVGs are commonly used in coronary artery bypass grafting (CABG) procedures. Over time, SVGs tend to degenerate and exhibit a predisposition to thrombus formation, which increases the risk of the no-reflow phenomenon in PCI procedures targeting these grafts.

Although no-reflow is known to occur more frequently in SVG interventions compared to native coronary arteries,^9^ studies exploring reliable and accessible predictors in this specific context are lacking.

To address this gap, the present study aims to investigate the relationship between AIP and the no-reflow phenomenon in patients with non-ST elevation myocardial infarction (NSTEMI) who undergo PCI for saphenous vein graft (SVG) lesions. We hypothesize that elevated AIP, as a marker of proatherogenic and proinflammatory metabolic status, may serve as an independent predictor of no-reflow in this high-risk population.

## Material

### Study Design and Patient Selection

This retrospective study was conducted at a single-center tertiary cardiovascular facility. The study population consisted of 287 patients who underwent PCI for SVG disease with NSTEMI.

A consecutive sampling method was used, including all eligible patients who met the inclusion criteria between February 2018 and December 2022.

The inclusion criteria were as follows: Patients with a history of CABG, patients presenting with NSTEMI undergoing PCI for significant SVG stenosis, and availability of pre-PCI lipid profile data, including triglyceride and high-density lipoprotein (HDL) cholesterol levels.

Exclusion criteria included patients with cardiogenic shock at presentation, those undergoing only percutaneous balloon angioplasty, prior stent implantation in the index vessel, severe valvular heart disease, stent restenosis or thrombosis, PCI for STEMI, or PCI for native coronary artery lesions instead of SVG.

The study was conducted in accordance with the principles of the Helsinki Declaration and was approved by the Ethics Committee of Istanbul University, Istanbul Faculty of Medicine (2025.01-06). Written informed consent was obtained from all participants included in the study

Sample Size Determination: In a study conducted by Ting-Ting Wu et al., which aimed to investigate the relationship between the AIP and the risk of CAD, as well as to determine whether AIP is a better predictor of CAD risk in postmenopausal women, the AIP was reported as 0.20 ± 0.27 in the CAD group and 0.10 ± 0.27 in the non-CAD group.^10^ Based on this study, it was assumed that a similar difference (Cohen’s d = ~0.3, representing a small-to-medium effect size) could also be observed between the no-reflow and non-no-reflow groups in our study. Under these assumptions, a power analysis determined that a minimum of 114 patients per group, for a total of 228 patients, would be required to achieve 80% power with a 5% margin of error.

All baseline demographic, clinical, and laboratory data were obtained from electronic medical records.

### Coronary angiography and PCI procedure

Coronary angiography and PCI procedures were performed according to standard institutional protocols. All patients received dual antiplatelet therapy, including 300mg aspirin and a P2Y12 inhibitor (clopidogrel 600mg, ticagrelor 180mg, or prasugrel 60mg) and 80 mg of atorvastatin before the intervention. A heparin bolus dose of 70–100 IU/kg was administered to each patient scheduled for angioplasty.

PCI strategies, including stent selection, use of predilatation or postdilatation, distal protection devices, or thrombectomy**,** were left to the operator’s discretion. TIMI (thrombolysis in myocardial infarction) flow grades were evaluated before and after the procedure to classify patients into no-reflow or normal reflow groups**.** The TIMI grading system defines grade 0 as the absence of antegrade flow beyond the occlusion, while grade 1 indicates minimal distal flow that fails to fully opacify the artery. Grade 2 represents delayed but complete opacification of the distal vessel, whereas grade 3 reflects normal perfusion with unrestricted flow. No-reflow was diagnosed when post-PCI TIMI flow remained below grade 3 in the absence of mechanical obstruction, such as dissection, residual stenosis, or vasospasm.

After stent implantation, patients were initially classified according to their immediate post-stenting TIMI flow grades. In cases of no-reflow, intracoronary pharmacologic therapies, including adenosine and glycoprotein IIb/IIIa inhibitors, epinephrine, or vasodilators, were applied according to operator discretion. Final TIMI flow grades were reassessed after these interventions, and patients achieving TIMI 3 flow were recorded accordingly. In eight patients, these interventions successfully restored final TIMI 3 flow. However, for statistical analysis and group classification, the TIMI flow grade assessed immediately after stent implantation and before additional pharmacological interventions was used. Thus, the no-reflow group includes patients regardless of any later improvement in TIMI flow following treatment.

### Measurement of lipid parameters

Blood samples were collected after an overnight fast before intervention and analyzed for lipid parameters using automated enzymatic assays. The total cholesterol, HDL cholesterol, low-density lipoprotein cholesterol (LDL-C), and triglyceride (TG) were assayed using an Abbott Diagnostics C8000i (Abbott, Germany) auto-analyzer with commercial kits. The AIP was calculated using the formula: log (TG/HDL-C).

### Statistical analysis

All statistical analyses were performed using SPSS software Version 21.0 for Windows (SPSS Inc., Chicago, IL, USA). The normality of data distribution was assessed using the Shapiro-Wilk test**.** Continuous variables were expressed as mean ± standard deviation (SD) for normally distributed data, and as median and interquartile range (IQR) for non-normally distributed data. Categorical variables were presented as frequencies and percentages**.**

Comparisons between the no-reflow and normal reflow groups were conducted using the following tests: Independent-samples t-test for normally distributed continuous variables, Mann-Whitney U test for non-normally distributed continuous variables, Chi-square test or Fisher’s exact test for categorical variables. A univariate logistic regression analysis was conducted to identify potential predictors of the no-reflow phenomenon. Multivariable logistic regression models were initially constructed using statistically significant variables (p < 0.05) in univariate analyses. Multicollinearity among predictors was assessed before model inclusion, and variables with significant collinearity were excluded. To reduce the risk of overfitting, a sensitivity analysis was performed using a parsimonious model comprising top predictors selected based on clinical relevance and univariate strength. Results were reported as odds ratios (ORs) with 95% confidence intervals (CIs)**.** A p-value < 0.05 was considered statistically significant for all analyses.

## Results

A total of 287 patients who underwent PCI for SVG disease were included in the study. These patients were categorized into two groups: normal reflow (70.7%) and no-reflow (29.3%). The baseline demographic, clinical, and laboratory characteristics of the study population are summarized in Table 1.

**Table 1:** – The baseline demographic, clinical, and laboratory characteristics of the study population

Variables	No-reflow (-) (n: 203)	No-reflow (+) (n: 84)	p value	% Difference (No-reflow - Reflow)
Age (years)	62.6±8.6	66.1±10	0.003	
Male sex, n (%)	173 (85.2%)	67 (79.8%)	0.255	
Current smoker, n (%)	44 (21.7%)	48 (57.1%)	0.061	
Hypertension, n (%)	155 (76.4%)	71 (84.5%)	0.124	
Diabetes mellitus, n (%)	80 (39.4%)	48 (57.1%)	0.006	17.17%
Dyslipidemia, n (%)	117 (57.6%)	48 (57.1%)	0.939	
Atrial fibrillation, n (%)	19 (9.4%)	15 (17.9%)	0.043	8.5%
Chronic heart failure, n (%)	39 (19.2%)	52 (61.9%)	<0.001	42.7%
Chronic kidney disease, n (%)	47 (23.2%)	31 (36.9%)	0.017	13.7%
Peripheral artery disease, n (%)	41 (20.2%)	25 (29.8%)	0.080	
COPD, n (%)	28 (13.8%)	9 (10.7%)	0.479	
History of stroke/TIA, n (%)	10 (4.9%)	19 (22.6%)	<0.001	17.7%
Previous MI, n (%)	97 (47.8%)	52 (61.9%)	0.029	14.1%
Previous PCI, n (%)	74 (36.5%)	39 (46.4%)	0.116	
LVEF, (%)	55 (45-60)	45 (35-50)	<0.001	
Hemoglobin (g/dl)	13.5±1.8	12.5±1.9	<0.001	
Total cholesterol (mg/dl)	192±54.5	202±57.3	0.075	
HDL-c (mg/dl)	39.7±9.2	35.3±9.9	0.001	
LDL-c, (mg/dl)	115.2±45.7	116±53.2	0.961	
Triglyceride (mg/dl)	163 (114-241)	205 (124-408)	0.002	
AIP	1.4 (1.04-2.00)	1.9 (1.23-2.50)	<0.001	

Variables with normal distribution were expressed as mean ± SD and compared with Student’s t-test, and Continuous variables without normal distribution are presented as median (interquartile range, IQR) and compared with Mann–Whitney U test. AIP: atherogenic index of plasma; COPD: chronic obstructive pulmonary disease; HDL-c: high-density lipoprotein cholesterol; LDL-c: low-density lipoprotein cholesterol; LVEF: left ventricular ejection fraction; MI: myocardial infarction; PCI: percutaneous coronary intervention; TIA: transient ischemic attack.

### Baseline characteristics

Compared to the normal reflow group, patients in the no-reflow group were older and had a higher burden of comorbidities, including diabetes, atrial fibrillation, chronic kidney disease, chronic heart failure, and prior stroke or transient ischemic attack (TIA). Laboratory analysis revealed lower HDL-C levels and higher triglyceride levels in the no-reflow group, resulting in a significantly elevated AIP.

### Angiographic and procedural characteristics

The angiographic and procedural characteristics are detailed in Table 2**.** The incidence of degenerated SVG was significantly higher in the no-reflow group. Additionally, the use of stents with larger diameters was more common in the no-reflow group, while stent length did not differ significantly **(**p = 0.559).

**Table 2:** – The coronary angiographic and procedural characteristics of patients

Variables	Normal reflow	No-reflow	p value	% Difference
Interval from operation to angiogram (years)	10.11 (5.06)	11.12 (4.98)	0.120	
Graft to the narrowed saphenous vein			0.910	
LAD, n (%)	13 (6.4%)	7 (8.3%)		
Diagonal, n (%)	22 (10.8%)	9 (10.7%)		
CX, n (%)	86 (42.24%)	37 (44%)		
RCA, n (%)	82 (40.4%)	31 (36.9%)		
Lesion site			0.074	
Osteal, n (%)	22 (10.8%)	15 (17.9%)		
Proximal, n (%)	55 (27.1%)	27 (32.1%)		
Middle, n (%)	63 (31%)	22 (26.2%)		
Distal, n (%)	53 (26.1%)	12 (14.3%)		
Preintervention TIMI flow grade			<0.001	
TIMI 0, n (%)	19 (9.4%)	22 (26.2%)		
TIMI 1, n (%)	14 (6.9%)	8 (9.5%)		
TIMI 2, n (%)	53 (26.1%)	30 (35.7%)		
TIMI 3, n (%)	117 (57.6%)	24 (28.6%)		
Postintervention TIMI flow grade				
TIMI 0, n (%)	0 (0%)	7 (8.3%)	<0.001	
TIMI 1, n (%)	0 (0%)	20 (23.8%)	<0.001	
TIMI 2, n (%)	0 (0%)	49 (58.3%)	<0.001	
TIMI 3, n (%)	203 (100%)	8 (9.5%)	<0.001	
Degenerated saphenous vein graft, n (%)	45 (22.2%)	53 (63.1%)	<0.001	40.9%
Thrombus, n (%)	40 (19.7%)	55 (65.5%)	<0.001	45.8%
Stent diameter (mm)	3.19 (0.51)	3.46 (0.59)	0.001	
Stent length (mm)	24.1 (11.81)	25.54 (13.59)	0.559	
Predilatation, n (%)	71 (35%)	38 (45.2%)	0.083	
Postdilatation, n (%)	48 (23.6%)	14 (16.7%)	0.191	
OAC usage, n (%)	15 (7.4%)	8 (9.5%)	0.544	
Glycoprotein IIb/IIIa inhibitor use, n (%)	35 (17.2%)	41 (48.8%)	<0.001	31.6%
Distal protection device use, n (%)	8 (3.9%)	4 (4.8%)	0.751	
Thrombectomy, n (%)	2 (1%)	4 (4.8%)	0.063	

CX: circumflex artery; LAD: left anterior descending artery; RCA: right coronary artery; OAC: oral anticoagulant; TIMI: Thrombolysis in Myocardial Infarction.

### Predictors of no-reflow

Univariate and multivariate logistic regression analyses were performed to identify predictors of no-reflow (Table 3). In the univariate analysis, age, diabetes mellitus, chronic kidney disease, chronic heart failure, history of stroke/TIA, atrial fibrillation, low left ventricular ejection fraction (LVEF), presence of degenerated SVG, thrombus burden, stent diameter, and triglyceride/HDL ratio were significantly associated with no-reflow.

**Table 3:** – Univariate and multivariate logistic regression analysis of predictors of the no-reflow phenomenon

	Univariate analyses		Multivariate analyses	
Variables	OR (95% CI)	p value	OR (95% CI)	p value
Age	1.044 (1.014-1.075)	0.004	1.032 (0.986-1.080)	0.176
Male sex	0.683 (0.354-1.320)	0.257		
Hypertension	1.691 (0.862-3.319)	0.127		
Diabetes Mellitus	2.050 (1.224-3.433)	0.006	2.127 (0.990-4.568)	0.053
Dyslipidemia	0.980 (0.586-1.639)	0.939		
Current smoker	1.712 (0.971-3.017)	0.063		
Chronic kidney disease	1.941 (1.120-3.366)	0.018	0.692 (0.297-1.613)	0.393
Chronic heart failure	6.883 (3.895-11.987)	<0.001		
Peripheral artery disease	1.674 (0.938-2.990)	1.674		
COPD	0.750 (0.338-1.666)	0.480		
History of stroke/TIA	5.642 (2.495-12.754)	<0.001	2.986 (0.999-8.925)	0.050
Atrial fibrillation	2.105 (1.013-4.374)	0.046	2.404 (0.743-7.775)	0.143
Previous MI	1.776 (1.056-2.985)	0.030	1.114 (0.500-2.485)	0.792
Previous PCI	1.511 (0.902-2.529)	0.110		
LVEF	0.916 (0.891-0.942)	<0.001	0.927 (0.892-0.964)	<0.001
Interval from operation to angiogram	1.040 (0.987-1.096)	0.140		
Degenerated SVG	6.320 (3.625-11.017)	<0.001	4.648 (2.143-10.082)	<0.001
Thrombus	8.150 (4.607-14.418)	<0.001	5.648 (2.143-10.082)	<0.001
*Stent diameter*	2.387 (1.467-3.885)	<0.001	3.724 (1.168-11.879)	0.026
*Stent length*	1.009 (0.998-1.031)	0.393		
Predilatation	1.577 (0.941-2.642)	0.084		
Postdilatation	0.646 (0.334-1.248)	0.193		
*Stent type*	0.526 (0.307-0.901)	0.019	1.520 (0.424-5.450)	0.521
AIP	2.332 (1.608-3.382)	<0.001	3.141 (1.636-6.030)	0.001

OR: Odds ratios; CI: confidence intervals; AIP: atherogenic index of plasma; COPD: chronic obstructive pulmonary disease; LVEF: left ventricular ejection fraction; MI: myocardial infarction; PCI: percutaneous coronary intervention; SVG: saphenous vein graft; TIA: transient ischemic attack.

In multivariate logistic regression analysis, degenerated SVG (OR: 4.648, 95% CI: 2.143–10.082, p < 0.001), thrombus presence (OR: 5.648, 95% CI: 2.143–11.879, p < 0.001), reduced LVEF (OR: 0.927, 95% CI: 0.892–0.964, p < 0.001), larger stent diameter (OR: 3.724, 95% CI: 1.168-11.879, p: 0.026) and elevated AIP (OR: 3.141, 95% CI: 1.636–6.030, p = 0.001), remained independent predictors of the no-reflow phenomenon. The central figure summarizes the main findings described above. To further address the risk of overfitting, a sensitivity analysis was conducted by repeating the multivariable logistic regression with the top six variables based on their univariate strength and clinical relevance (degenerated SVG, thrombus, reduced LVEF, AIP, and history of stroke/TIA). This parsimonious model produced consistent results, reaffirming the robustness of our findings (Table 4).

**Table 4:** – Sensitivity Analysis of Independent Predictors of the No-Reflow Phenomenon

Multivariate analyses		
Variables	OR (95% CI)	p value
History of stroke/TIA	3.130 (1.102-8.889)	0.032
LVEF	0.921 (0.888-0.954)	<0.001
Degenerated SVG	4.814 (2.325-9.970)	<0.001
Thrombus	4.978 (2.369-10.460)	<0.001
Stent diameter	2.460 (1.291-4.686)	0.006
AIP	2.531 (1.451-4.414)	0.001

OR: Odds ratio; CI: confidence interval; AIP: atherogenic index of plasma; LVEF: left ventricular ejection fraction; SVG: saphenous vein graft; TIA: Transient ischemic attack.

## Discussion

In this study, we investigated the relationship between the no-reflow phenomenon occurring during SVG intervention in NSTEMI patients with a history of CABG and the AIP, calculated as the log (triglyceride/HDL ratio). Our multivariate analysis demonstrated that degenerated SVG, the presence of thrombus, reduced LVEF, and elevated AIP were independent predictors of NR. The ease of obtaining the triglyceride/HDL ratio in routine clinical practice suggests that this parameter may serve as a useful marker for predicting microvascular dysfunction and the risk of no-reflow.

Our findings are consistent with other studies in the literature highlighting the association of AIP with CAD risk, plaque progression, and the no-reflow mechanism driven by microvascular injury. Studies conducted in STEMI patients have shown that AIP independently predicts no-reflow and offers superior predictive power compared to other lipid parameters.^11-13^ The relatively straightforward calculation of AIP and similar lipid parameters in clinical settings further underlines the importance of this indicator in estimating no-reflow risk.

AIP is calculated as the logarithm of the ratio between triglycerides (TG) and HDL-C, serving as an important biomarker of atherosclerotic processes in the vascular wall. It has emerged as a promising tool for evaluating CAD risk, disease severity, and prognosis.^11^ Studies indicate that AIP is strongly associated with multiple pro-atherogenic mechanisms, including elevated remnant lipoproteins, increased cholesterol esterification, and insulin resistance, which together promote endothelial dysfunction, plaque vulnerability, and a pro-inflammatory state—key contributors to the no-reflow phenomenon.^14-16^Elevated AIP reflects an imbalance between atherogenic triglyceride-rich lipoproteins and protective HDL particles. This imbalance promotes the formation of small, dense LDL particles, which are more prone to oxidation, leading to endothelial injury and inflammation. Additionally, increased triglyceride levels contribute to a greater burden of remnant lipoproteins, which infiltrate the vascular intima and stimulate local inflammatory responses. These processes not only enhance the progression of atherosclerosis but also increase the risk of distal embolization and microvascular damage. Moreover, low HDL levels reduce reverse cholesterol transport and the endothelial repair mechanism, further compromising coronary microcirculation. Collectively, these interrelated mechanisms offer a clear pathophysiological explanation for how elevated AIP may predispose patients to no-reflow during PCI.

Patients with a history of CABG are explicitly classified as very high cardiovascular risk, which recommends an LDL-C target of <55 mg/dL (1.4 mmol/L) and a ≥50% reduction from baseline. In cases of a second vascular event within two years, a more stringent goal of <40 mg/dL (1.0 mmol/L) may be considered.^17^ Robust genetic, epidemiological, and randomized clinical trial evidence has established a direct causal relationship between elevated LDL-C and atherosclerotic cardiovascular disease, including graft failure.^18^ Accordingly, intensive lipid-lowering therapy, primarily with high-dose statins and adjunctive agents such as ezetimibe or PCSK9 inhibitors, is essential for secondary prevention.^17,19,20^ In our study cohort, the mean LDL-C levels remained above target despite prior CABG, suggesting either suboptimal treatment intensity or poor adherence. This persistent atherogenic burden may have contributed to impaired microvascular perfusion and increased risk of adverse outcomes. These findings highlight the critical importance of achieving guideline-recommended lipid targets in the context of secondary prevention, particularly among patients who have undergone surgical revascularization.

In the context of cardiometabolic-based chronic disease, impaired lipid metabolism and insulin resistance play major roles in atherogenesis, emphasizing the value of lipoprotein-based indicators like AIP in cardiometabolic risk assessment.^14^ Another investigation examining the relationship between AIP, insulin resistance, and type 2 diabetes revealed a non-linear association, suggesting that elevated AIP may be an important risk marker, especially for diabetes and insulin resistance that elevated AIP may be an important risk marker, particularly for diabetes and insulin resistance.^15^ Moreover, high AIP levels have been reported to increase remnant lipoproteins and cholesterol esterification, thus accelerating atherogenic changes in the vascular wall.^21^ Collectively, these findings demonstrate that AIP is strongly associated with insulin resistance, remnant lipoproteinemia, and increased cholesterol esterification—factors that accelerate atherosclerosis—and support its effective use in clinical practice for cardiometabolic risk evaluation.

No-reflow arises from functional and structural alterations in the coronary microcirculation, explained by four main mechanisms: distal embolization, ischemic injury, reperfusion injury, and individual susceptibility.^22^ Specifically, the embolization of material from atherosclerotic plaques into the distal vascular bed promotes microinfarcts and inflammation, facilitating no-reflow.^23^

Prolonged ischemia increases cell death and edema while reducing nitric oxide production, which further enhances vascular permeability through vascular endothelial growth factor (VEGF).^24^

Reperfusion injury aggravates microvascular damage through neutrophil infiltration, cytokine release, and oxidative stress. Preexisting endothelial dysfunction or genetic predispositions can also heighten susceptibility to no-reflow.

Inflammation plays a particularly critical role in this process, as it is widely recognized as a key contributor to both CAD pathophysiology and the development of no-reflow. Various studies have shown that higher C-reactive protein (CRP) levels and lower HDL-C concentrations are associated with no-reflow, highlighting the prominent impact of an inflammatory state in this phenomenon.^25^ Systematic reviews also indicate that the remnant particles produced during the lipolysis of triglyceride-rich lipoproteins can provoke vascular inflammation, thereby advancing atherosclerosis.^26^ Therefore, the higher incidence of no-reflow observed in patients with elevated AIP may largely be attributed to underlying inflammatory processes. Since AIP can be easily measured in routine practice, it shows promise as a practical indicator for predicting the risk of no-reflow associated with both microvascular dysfunction and inflammation.

In our study, apart from the elevated AIP, we found that degenerated SVG, the presence of thrombus, stent diameter, and reduced LVEF emerged as independent predictors of the no-reflow phenomenon. Degenerated grafts likely reflect advanced atherosclerotic changes and increased plaque vulnerability, predisposing to distal embolization and microvascular obstruction—a finding that aligns with previous reports demonstrating the adverse impact of graft degeneration on reperfusion outcomes. Similarly, thrombus formation within the graft can lead to distal embolization, microinfarcts, and subsequent impairment of microvascular perfusion, as also highlighted by other studies in similar clinical settings. Reduced LVEF, on the other hand, not only indicates the severity of myocardial damage but also suggests an impaired microcirculatory reserve, thereby increasing the risk for no-reflow. These associations underline the multifactorial nature of the no-reflow phenomenon and the importance of integrating both lipid-related and hemodynamic parameters in risk stratification, as supported by the literature.^8^

Additionally, our findings revealed that a larger stent diameter was independently associated with no-reflow. One possible explanation is that larger stents are often required for severely degenerated or ectatic saphenous vein grafts, which tend to have higher thrombus burden and friable atherosclerotic plaques. These anatomical features increase the risk of distal embolization and microvascular obstruction, key mechanisms contributing to the development of no-reflow. This observation is consistent with previous reports indicating that lesion complexity and plaque burden are critical contributors to microvascular perfusion impairment during PCI in SVG lesions.^22,24^

Limitations and Strengths: Despite these promising insights, our study has certain limitations. The retrospective design and single-center nature may restrict the generalizability of the findings, and potential confounding factors inherent to observational research could not be entirely ruled out. Nevertheless, the study’s strengths include the comprehensive evaluation of routinely available clinical and angiographic parameters, which enhances its applicability in everyday practice. Another limitation of our study is the relatively low use of distal protection devices during SVG interventions, despite guideline recommendations supporting their benefit in selected cases. This may have contributed to the high incidence of no-reflow by allowing more frequent distal embolization. The limited use of these devices was largely due to local healthcare constraints and cost-related barriers in our country. Although the ACC/AHA guidelines recommend embolic protection devices (EPDs) for SVG interventions (Class I indication), real-world use remains low. Data from the NCDR Cath Registry (~21% usage) showed no significant reduction in adverse outcomes and even higher procedural complications, such as no-reflow and perforation, in the EPD group. These findings highlight that EPD use should be individualized based on thrombus burden, lesion complexity, and operator experience.^27^ In our center, cost constraints and limited availability further restricted EPD use to selected high-risk cases. Additionally, although all patients had a history of prior coronary revascularization, the mean LDL-C levels in our cohort remained above the recommended target of 50 mg/dL, particularly for secondary prevention. This suboptimal lipid control may reflect underuse or nonadherence to intensive statin therapy and could have influenced microvascular dysfunction and no-reflow risk. Another limitation is the absence of propensity score matching (PSM) to adjust for baseline differences. Due to the limited number of no-reflow cases, applying PSM could have significantly reduced the sample size and statistical power. Future studies with larger populations should consider using PSM or other advanced matching techniques to validate these findings.

Moreover, by confirming the independent predictive value of readily obtainable markers such as AIP, degenerated grafts, thrombus presence, and LVEF, our work contributes valuable evidence toward improving risk assessment and guiding preventive strategies for the no-reflow phenomenon in high-risk patient populations.

Future studies should ideally adopt prospective, multi-center designs to validate our findings and explore potential interventions that might mitigate these risk factors. Investigating strategies to improve graft integrity and reduce thrombus burden could be particularly valuable. Moreover, assessing the prognostic value of AIP in broader patient populations and determining optimal cut-off values for clinical use would be beneficial in refining risk stratification and tailoring treatment approaches.

Given its simplicity and accessibility, AIP may serve as a useful prognostic marker not only in SVG interventions but also in broader populations, such as those with native coronary artery disease or chronic coronary syndromes. Further prospective studies are needed to validate its role and establish clinically meaningful cut-off values in these settings.

## Conclusion

In summary, our study demonstrates that elevated AIP, degenerated SVGs, thrombus presence, stent diameter, and reduced LVEF are independently associated with the no-reflow phenomenon in a carefully selected patient population. These findings provide additional insights into the multifactorial nature of no-reflow and suggest that routinely available clinical and angiographic parameters may aid in its early identification. However, given the retrospective, single-center design of our study, further research is needed to confirm these associations and explore the impact of targeted interventions on improving clinical outcomes in high-risk patients.

Ethics approval and consent to participate: This study was approved by the Ethics Committee of the Istanbul University, Istanbul Faculty of Medicine (2025.01-06). All the procedures in this study were in accordance with the 1975 Helsinki Declaration, updated in 2013. Informed consent was obtained from all participants included in the study.
